# “Green” Prussian Blue Analogues as Peroxidase Mimetics for Amperometric Sensing and Biosensing †

**DOI:** 10.3390/bios11060193

**Published:** 2021-06-10

**Authors:** Galina Z. Gayda, Olha M. Demkiv, Yanna Gurianov, Roman Ya. Serkiz, Halyna M. Klepach, Mykhailo V. Gonchar, Marina Nisnevitch

**Affiliations:** 1 Institute of Cell Biology, National Academy of Sciences of Ukraine, 79005 Lviv, Ukraine; demkivo@nas.gov.ua (O.M.D.); roman.serkiz@lnu.edu.ua (R.Y.S.); gonchar@cellbiol.lviv.ua (M.V.G.); 2 Faculty of Veterinary Hygiene, Ecology and Law, Stepan Gzhytskyi National University of Veterinary Medicine and Biotechnologies, 79000 Lviv, Ukraine; 3 Department of Chemical Engineering, Ariel University, Kyriat-ha-Mada, Ariel 4070000, Israel; yannag@ariel.ac.il; 4 Faculty of Biology and Natural Sciences, Drohobych Ivan Franko State Pedagogical University, 82100 Drohobych, Ukraine; pavlishko@yahoo.com

**Keywords:** artificial enzymes, green synthesis, hexacyanoferrates of transition and noble metals, peroxidase mimetic, amperometric (bio)sensor, glucose oxidase, glucose analysis

## Abstract

Prussian blue analogs (PBAs) are well-known artificial enzymes with peroxidase (PO)-like activity. PBAs have a high potential for applications in scientific investigations, industry, ecology and medicine. Being stable and both catalytically and electrochemically active, PBAs are promising in the construction of biosensors and biofuel cells. The “green” synthesis of PO-like PBAs using oxido-reductase flavocytochrome *b*_2_ is described in this study. When immobilized on graphite electrodes (GEs), the obtained green-synthesized PBAs or hexacyanoferrates (gHCFs) of transition and noble metals produced amperometric signals in response to H_2_O_2_. HCFs of copper, iron, palladium and other metals were synthesized and characterized by structure, size, catalytic properties and electro-mediator activities. The gCuHCF, as the most effective PO mimetic with a flower-like micro/nano superstructure, was used as an H_2_O_2_-sensitive platform for the development of a glucose oxidase (GO)-based biosensor. The GO/gCuHCF/GE biosensor exhibited high sensitivity (710 A M^−1^m^−2^), a broad linear range and good selectivity when tested on real samples of fruit juices. We propose that the gCuHCF and other gHCFs synthesized via enzymes may be used as artificial POs in amperometric oxidase-based (bio)sensors.

## 1. Introduction

Artificial enzymes are stable and low-cost mimetics of natural enzymes. The search for effective novel artificial enzymes, especially nanozymes, and the development of simple methods for their synthesis and characterization, as well as the selection of novel branches for their application, are currently challenging problems in different fields of biotechnology, industry, and medicine [1,2,3,4,5,6,7,8,9].

Peroxidase (PO) mimetics are the most frequently investigated artificial enzymes [10,11,12]. One of the well-known effective PO-like artificial enzymes is Prussian blue (PB) or iron(III) hexacyanoferrate (FeHCF). PB is a member of a well-documented family of synthetized coordination compounds with an extensive 300-year history [13,14,15,16]. PB and its analogs (PBAs) are cheap and easy to synthesize, environmentally friendly, and have potential applications for basic research and industrial purposes [12,13,14,15,16,17,18] in a large variety of fields, particularly in medicine [13,19,20,21,22,23]. Despite their multifunctionality, PBAs have complicated compositions, which are largely dependent on the synthesis methods and storage conditions [14,15,16]. Insoluble PB can be described by the formula Fe_4_[Fe(CN)_6_]_3_, while KFe[Fe(CN)_6_] corresponds to a colloidal solution of PB. The general formula of hexacyanoferrate (HCF) is M_k_[Fe(CN)_6_] × H_2_O, where M is a transition metal [14,15].

Due to their capability to insert various ions as counter-ions during the redox process, PB and PBAs have attracted increasing interest as electrode materials for energy storage in fuel cells [15,24,25]. Having remarkable super-magnetic properties, redox and PO-like activities, PBAs are widely applied in bioreactors for detoxification of dangerous chemicals [13,17,26], in molecular magnets, and in optical and electrochemical biosensors [12,13,14,15,16,24,27].

The first report of electrochemical reduction of H_2_O_2_ on PB-modified electrodes was published by Itaya in 1984 [28]. In 2000, Karyakin named PB as an “artificial PO” and published numerous reports concerning PB-based amperometric biosensors (ABSs) [24,29,30,31,32,33]. Numerous other scientific groups, especially from China, have also worked diligently on this problem [18,19,20,21,22,23,34,35,36,37].

PBAs are usually obtained via various techniques, including chemical [12,13,14,15,16] and biological methods [38,39,40]. The biosynthesis of materials using plants, microorganisms and their metabolites as biosurfactants can be related to “green synthesis (GS) [41,42,43,44]. Purified enzymes were also shown to be capable of reducing metal ions to obtain metallic nanoparticles [40,45,46].

The application of green-synthesized PBAs (gPBAs or gHCFs) for the construction of ABSs is not yet well documented. The main advantages of green-synthesized nanomaterials (gNMs) are the low energy cost of their synthesis, lack of toxic chemicals, simplicity of procedure, high adaptability of the synthesized gNM, and the presence of functional groups on their surface. The latter is promising for simple immobilization of bioorganic molecules, including enzymes, during biosensor construction [40,47]. If the gNM has additional catalytic properties, it plays a dual role in biosensors, simultaneously serving as the carrier of bio-elements and as the enzyme mimetic (nanozyme).

In our previous research, we reported obtaining gHCFs of transition metals using the purified yeast enzyme flavocytochrome *b*_2_ (Fc*b*_2_; L-lactate: ferricytochrome c oxidoreductase, EC 1.1.2.3). The structure, size, composition, electro-catalytic properties, electro-mediator activity, and PO-like properties of the obtained gHCFs, which were synthesized via an enzyme and incorporated with it, were characterized. A more detailed study was performed on copper hexacyanoferrate (gCuHCF or gCuPBA), which was found to be the most effective PO mimetic. When immobilized on a GE, the gCuHCF under special pH conditions and working potential gave the intrinsic amperometric response to hydrogen peroxide. We demonstrated that the synthesized gCuHCF may be successfully used as an artificial PO for sensor analysis of hydrogen peroxide in a real disinfectant sample [40].

In the current work, we describe in more detail the synthesis and characteristics of new gHCFs of transition and noble metals with PO-like activity, an additional structural study of the most effective gCuHCF, development of an improved and highly sensitive ABS using glucose oxidase (GO) and gCuHCF, and testing of the constructed GO/gCuHCF ABS for glucose analysis in real samples of fruit juices.

## 2. Materials and Methods

### 2.1. Reagents

Potassium ferricyanide (K_3_Fe(CN)_6_), iron(III) chloride (FeCl_3_ × 4H_2_O), copper(II) sulfate (CuSO_4_), Cerium(IV) sulfate tetrahydrate Ce(SO_4_)_2_ × 4H_2_O, palladium chloride (PdCl_3_), cobalt(II) chloride (CoCl_2_ × 6H_2_O), zinc(II) sulfate (ZnSO_4_), manganese(II) chloride (MnCl_2_ × 4H_2_O), cadmium(II) chloride (CdCl_2_), neodymium(III) chloride (NdCl_3_), 2,2′-azinobis (3-ethylbenzothiazoline-6-sulfonate) diammonium salt (ABTS), o-dianisidine, hydrogen peroxide (H_2_O_2_, 30%), sodium ethylenediaminetetraacetate (EDTA), sodium L-lactate, Nafion (5% solution in 90% low-chain aliphatic alcohols) and all other reagents and solvents used in this work were purchased from Sigma-Aldrich (Steinheim, Germany). All reagents were analytical grade and were used without further purification. All solutions used ultra-pure water prepared with the Milli-Q^®^ IQ 7000 Water Purification system (Merck KGaA, Darmstadt, Germany).

### 2.2. Enzymes

Flavocytochrome *b*_2_ (Fc*b*_2_) was isolated from the yeast *Ogataea (Hansenula) polymorpha 356* and purified, as described earlier [48,49]. The Fc*b*_2_ (20 U·mg^−1^) was stored at −10 °C in a suspension of 70% ammonium sulfate, prepared with 50 mM phosphate buffer, pH 7.5, containing 1 mM EDTA and 0.1 mM dithiothreitol. To prepare a fresh solution, the enzyme was precipitated from the suspension by centrifugation (10,000 rpm, 10 min, 4 °C) and dissolved in 50 mM phosphate buffer, pH 7.5, up to 50 U·mL^−1^. An assay of Fc*b*_2_ activity in solution was performed as described earlier [48,49]. One unit of the enzyme activity was defined as the amount of enzyme that oxidizes 1 μmol of L-lactate in 1 min under standard assay conditions (20 °C; 30 mM phosphate buffer, pH 7.5; 0.33 M L-lactate; 0.83 mM K_3_Fe(CN)_6_; 1 mM EDTA).

A commercial lyophilized horseradish peroxidase (PO or HRP, EC 1.11.1.7) from *Armoracia rusticana* (Aster, Lviv, Ukraine) with 600 U·mg^−1^ activity was dissolved in 20 mM phosphate buffer, pH 6.0, up to 400 U·mL^−1^.

A commercial lyophilized glucose oxidase (GO, EC 1.1.3.4) from *Asperigillus niger* (Sigma, St. Louis, MO, USA) with an activity of 100,000 U·g^−1^ in a solid form was dissolved in 20 mM phosphate buffer, pH 6.0, up to a concentration of 0.1 mg·mL^−1^. GO activity was assayed in a reaction mixture containing 0.16 mM *o*-dianisidine, 1.61% (*w*/*v*) glucose and 2 U mL^−1^ of PO in 50 mM sodium acetate buffer (NaOAc), pH 5.0, as described earlier [50].

### 2.3. Synthesis of Hexacyanoferrates

**Figure 1:**
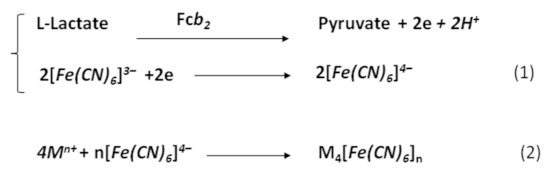
Scheme of green hexacyanoferrate synthesis using flavocytochrome *b*_2_ (Fc*b*_2_) in enzymatic (**1**) and chemical (**2**) reactions; M—metal.

Synthesis of gHCF was carried out according to the scheme presented in Figure 1 [40]. A reaction mixture containing 6 mM K_3_[Fe(CN)_6_], 20 mM sodium lactate, 0.03–0.15 U mL^−1^ Fc*b*_2_ in 50 mM phosphate buffer, pH 8.0, was prepared and incubated at 37 °C for 30 min. Formation of gHCF was initiated by the addition of salt to a final concentration of 10–100 mM.

To obtain chemically synthesized HCFs (chHCFs), a solution of 6 mM K_3_Fe(CN)_6_ and 60 mM transition metal salt in 50 mM phosphate buffer, pH 8.0, was mixed with H_2_O_2_, added dropwise up to 100 mM. After 0.5–10 min incubation, the resulting mixture was fractionated by centrifugation at 13,000 rpm for 1 min, and the precipitate was resuspended in water. The centrifugation–redispersion procedure was repeated 2–4 times. The obtained HCFs were resuspended in water and kept at +4 °C until used.

### 2.4. Characterization of the Synthesized HCFs

#### 2.4.1. Optical Properties

The optical properties of the synthesized HCFs, their concentrations and PO-like activities were characterized using a Shimadzu UV1650 PC spectrophotometer (Kyoto, Japan).

#### 2.4.2. Scanning Electron Microscopy (SEM)

Morphological analyses of the samples were performed using a SEM microanalyzer REMMA-102-02 (Sumy, Ukraine). The samples of different dilutions (2 μL) were dropped onto the surface of a silicon wafer and dried at room temperature. The distance from the last lens of the microscope to the sample (WD) ranged from 17.1 to 21.7 mm. The accelerator voltage was in the range of 20 to 40 eV.

#### 2.4.3. FTIR Analysis

The infrared spectra were prepared using the KBr pellet technique, by thoroughly mixing 3 μL of a particle suspension with 0.2 g of KBr and pressing at 5 ton_f_ using a hydraulic press (Carver^®^ Inc., Wabash, IN, USA). The samples were dried in a desiccator overnight and analyzed by the Spectrum^TM^ One FTIR Spectrometer (Perkin Elmer, Waltham, MA, USA) at room temperature in the 4000–400 cm^−1^ range at an operation number of 20 scans, a resolution of 4.0 cm^−1^, and a scanning interval of 1 cm^−1^.

#### 2.4.4. Particle Counter Analysis

Particle concentration was measured using a particle counter (Spectrex Corp., Redwood, CA, USA) in a round-shaped 150 mL transparent glass bottle with a wall thickness of 2 mm. A total of 10 μL of the sample was added to the bottle with 99 mL of water (HPLC grade, Bio-Lab Ltd., Jerusalem, Israel) under continuous stirring. Particle counting was performed with a laser diode at a wavelength of 650 nm.

#### 2.4.5. Dynamic Light Scattering (DLS) Analysis and Zeta-Potential Measurements

The DLS analysis and zeta-potential measurements were performed using a Litesizer 500 type BM10 instrument (Anton Paar GmbH, Graz, Austria) at 25 °C. For measurement of hydrodynamic diameters, the samples were diluted to 1:150, 1:300, and 1:600 with HPLC-grade water, placed into a semi-micro quartz cell, and analyzed using a laser at a wavelength of 660 nm and a side scatter of 90°. Zeta-potential was measured in diluted colloidal solutions at a particle concentration of 1.33 × 10^4^ mL^−1^, which was determined as described in Section 2.4.4. The solutions were injected into an omega-shaped cuvette and analyzed at an operating voltage of 200 V.

#### 2.4.6. X-ray Diffraction (XRD) Analysis

The phase composition of synthesized particles was studied by XRD analysis using a Rigaku SmartLab SE X-ray powder diffractometer with Cu Kα radiation (λ = 0.154 nm) for phase identification. Full-pattern identification was carried out by a SmartLab Studio II software package, version 4.2.44.0 from the Rigaku Corporation (Tokyo, Japan). Materials identification and analysis were performed by the ICDD base PDF-2 Release 2019 (Powder Diffraction File, ver. 2.1901). XRD patterns were obtained using 40 kV, 30 mA by Θ/2Θ (Bragg-Brentano geometry) in the 2Θ range of 10–90° (step size 0.03° and speed 4°/min). The crystallite size was calculated using quantitative analysis based on the Halder–Wagner method, with the help of the program Powder XRD plugin of SmartLab Studio II x64 v4.2.44.0.

### 2.5. Assay of Enzyme-Like Activities of the Synthesized HCFs in Solution

PO-like activity of the HCFs was measured by the colorimetric method, with *o*-dianisidine and ABTS as chromogenic substrates in the presence of H_2_O_2_. One unit (U) of PO-like activity was defined as the amount of HCF releasing 1 µmol H_2_O_2_ per 1 min at 30 °C under standard assay conditions. To estimate special enzyme-like activity (U/mg), the HCFs were dried. The tested solution/suspension was prepared by weighing the solid substance and adding water until the needed concentration was obtained.

The assay of PO-like activity with *o*-dianisidine: 10 μL of the aqueous suspension of HCF (1 mg mL^−1^) was incubated in a glass tube with 1 mL of 0.17 mM o-dianisidine in water (as a control), and with the same substrate in the presence of 8.8 mM H_2_O_2_ (as a substrate for PO). The addition of NPs to the substrate stimulated the development of an orange color over time, indicating an enzymatic reaction. The enzyme-mimetic activity could be assessed qualitatively with the naked eye and was measured quantitatively with a spectrophotometer. After incubation for an exact time (1–10 min) at 30 °C, and upon the appearance of the orange color, the reaction was stopped by the addition of 0.26 mL 12 M HCl. The generated color was determined at 525 nm using a spectrophotometer. The millimolar extinction coefficient (ε) of the resulting pink dye in the acidic solution was 13.38 mM^−1^·cm^−1^.

The assay of PO-like activity with ABTS: 10 μL of the aqueous suspension of HCF was incubated in a 1 mL quartz cuvette with 1 mM ABTS in water (as a chromogenic substrate for oxidase), and with the same substrate in the presence of 12 mM H_2_O_2_ (as a substrate for PO-like HCF). The addition of HCF to the corresponding substrate (ABTS for oxidase-like HCF, ABTS with H_2_O_2_ for PO-like HCF) stimulated the development of a green color over time, indicating an enzymatic reaction. The enzyme-mimetic activity could be assessed with the naked eye and was measured quantitatively with a spectrophotometer. The speed of appearance of a green color was monitored at 420 nm over time using a spectrophotometer, thus enabling calculation of the enzyme-like activity. The coefficient ε of the resulting green dye was 36.0 mM^−1^·cm^−1^.

### 2.6. Sensor Evaluation

#### 2.6.1. Apparatus and Measurements

The amperometric sensors were evaluated using constant–potential amperometry in a three-electrode configuration with an Ag/AgCl/KCl (3 M) reference electrode, a Pt-wire counter electrode, and a working graphite electrode. Graphite rods (type RW001, 3.05 mm diameter) from Ringsdorff Werke (Bonn, Germany) were sealed in glass tubes using epoxy glue for disk electrode formation. Before sensor preparation, the graphite electrode (GE) was polished on emery paper and on a polishing cloth using decreasing sizes of alumina paste (Leco, Germany). The polished electrodes were rinsed with water in an ultrasonic bath.

Amperometric measurements were carried out using a potentiostat CHI 1200 A (IJ Cambria Scientific, Burry Port, UK) connected to a personal computer, performed in a batch mode under continuous stirring in an electrochemical cell with a 20 mL volume at 25 °C.

All experiments were carried out in triplicate trials. Analytical characteristics of the proposed electrodes were statistically processed using the OriginPro 8.5 software. Error bars represent the standard error derived from three independent measurements. Calculation of the apparent Michaelis–Menten constants (*K_M_^app^*) was performed automatically by this program according to the Lineweaver–Burk equation.

#### 2.6.2. Immobilization of HCFs and the Enzyme onto Electrodes

The HCFs and enzymes were immobilized on the GEs using the physical adsorption method.

For the development of the HCF or PO-based electrode, 5 μL of HCF or 5 μL of enzyme solution was dropped onto the surface of bulk GEs. After drying for 10 min at room temperature, the layer of HCF or enzyme on the electrode was covered with 10 μL of Nafion. The modified electrodes were rinsed with corresponding buffers and kept in these buffers at 4 °C until used.

To fabricate the glucose oxidase (GO)-based biosensor, 8 μL of GO solution (5 U/mL) was dropped onto the dried surface of the gCuHCF-modified GE. The dried composite was covered by a Nafion membrane. The coated bioelectrode was rinsed with water and stored in 50 mM phosphate buffer, pH 6.0, until used.

## 3. Results and Discussion

### 3.1. gHCFs-Modified Electrodes for Hydrogen Peroxide Sensing

According to the literature, chemically synthesized HCFs (chHCFs) of Fe (III), Mn (II) and Cu (II) demonstrate significant PO-like activity in solution and on electrodes [13,14,15,16,29,31]. In the current work, several gHCFs were obtained via Fc*b*_2_ from the corresponding salts (Fe, Cu, Pd, Ce, Mn, et al.) and from K_4_Fe(CN)_6_, a product of K_3_Fe(CN)_6_ reduction by L-lactate in the presence of an enzyme (Figure 1). Our first task was to screen the obtained gPBAs for their sensitivity to H_2_O_2_ on amperometric graphite electrodes (GEs) and to select the best compounds as PO mimetics. For this purpose, the optimal conditions for the amperometric experiments were investigated. The amperometric characteristics of the control GE (not modified with gHCF) as a chemosensor for H_2_O_2_ were tested using cyclic voltammetry (CV) analysis. Selection of the optimal pH, working potential and scan rate was carried out according to the CV results (data not shown).

Under the experimentally chosen optimal conditions (50 mM NaOAc buffer, pH 4.5 and −50 mV as the working potential), numerous electrodes modified with the synthesized HCFs were screened for their ability to decompose hydrogen peroxide. A low working potential is necessary in order to avoid the effect of possible interfering substances on the electrode’s response in the presence of oxygen. This requirement is relevant for the construction of biosensors and their exploitation for the analysis of real samples (food products, biological liquids, and others).

**Figure 2:**
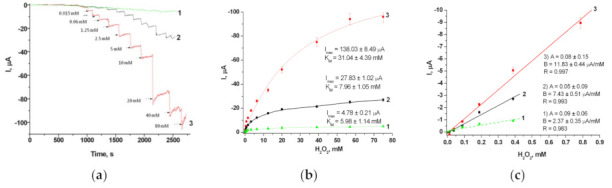
Amperometric characteristics of the modified electrodes: chronoamperograms (**a**), dependences of the response on increasing concentrations of H_2_O_2_ (**b**), and calibration graphs (**c**) for PO/GE (1), gFeHCF/GE (2), and gCuHCF/GE (3). Conditions: working potential −50 mV versus Ag/AgCl (reference electrode), 50 mM NaOAc buffer, pH 4.5 at 23 °C.

The electrocatalytic activities of the synthesized HCFs immobilized on the surface of GEs were tested by CV and chronoamperometry, as described in Section 2.6.1. The amperometric responses of different HCF/GEs to the added H_2_O_2_ were compared. Following the chronoamperograms, calibration curves were plotted for H_2_O_2_ determination by the developed electrodes (Figure 2 and Figure S1). The linear ranges and sensitivities of the electrodes modified with HCF were calculated. The analytical characteristics of the developed HCF/GEs, as deduced from the graphs (Figure 2 and Figure S1), are summarized in Table 1.

Modification of GEs with the gHCFs improved the efficiency of electron transfer due to the increase in the electrochemically accessible electrode surface area. It is worth mentioning that in comparison to native PO, several gHCF/GEs displayed higher current responses (*I_max_*) to H_2_O_2_ at substrate saturation and higher sensitivities (Table 1). The enhancement of current outputs and sensitivities of the electrodes modified with other gHCFs were insignificant. Thus, gCuHCF, gFeHCF, gPdHCF and gCeHCF, when immobilized on graphite electrodes, demonstrated higher PO-like activities in comparison with other gHCFs, as well as with native PO and chemically synthesized chCuHCF (Table 1, Figure 2 and Figure S1). For the most effective electrode (gCuHCF/GE), the current response (*I_max_*) to H_2_O_2_ at substrate saturation was five-fold higher, and the sensitivity was 29-fold higher than those of the PO/GE (Table 1).

As seen, the results presented in Table 1 supported the gCuHCF/GE as the most effective PO mimetic. It was therefore studied in more detail.

Many of the reported H_2_O_2_-sensitive PBA-based sensors have sensitivities similar to the developed gCuHCF/GE sensor (1620 A M^−1^m^−2^) [40]. For example, a PB-modified glassy carbon electrode (GCE) demonstrated sensitivity of 2000 A M^−1^m^−2^ [51], MnPBA/GCE—1472 A M^−1^m^−2^ [37]. Graphite-paste electrodes, modified with Ni-FePBA and Cu-FePBA, showed sensitivities of 1130 and 2030 A M^−1^m^−2^, respectively [52]. Diamond-boron doped (DBD) electrodes, modified with PB and Ni-FePBA, demonstrated sensitivities of 2100 and 1500 A M^−1^m^−2^, respectively [53].

Other H_2_O_2_-sensitive sensors that contain PBA, coupled with other nanomaterials (carbon, graphene, natural polysaccharides, or synthetic polymers), demonstrated significantly higher sensitivities (from 3–5-fold [16,27,29,32,33,34] up to 300-fold [54]) compared with the gCuHCF/GE. The main peculiarities of the described sensors were high stability, sensitivity, and selectivity towards H_2_O_2_ in extra-wide linear ranges. These properties led to the successful use of the PBAs in oxidase-based biosensors [29,30,31,32,33,35,36,40,51,54,55,56].

The results obtained by us indicated that the gCuHCF and other gHCFs may have a potential for use as PO-like composites for the construction of amperometric oxidase-based biosensors.

### 3.2. Study of Structure, Morphology, and Size of the gCuHCF Composite

The size, morphology, and composition of any materials, especially of NPs, are considered as their basic parameters. A number of noninvasive label-free methods were developed for the characterization of different materials: scanning electron microscopy (SEM), transmission electron microscopy (TEM), dynamic light scattering (DLS), Fourier transform infrared spectroscopy (FTIR), X-ray diffraction (XRD) analysis, Raman spectroscopy, atomic force microscopy (AFM) and other approaches. FTIR spectroscopy allows rapid acquisition of a biochemical fingerprint of the sample under investigation, giving information on its main biomolecule content. DLS allows the rapid determination of diffusion coefficients and also provides information on relaxation time distribution for the macromolecular components of complex systems and their hydrodynamic diameters. XRD provides information regarding the crystallographic structure**** of a material**** based on incident X-ray irradiation of the material and measurement of scattering angles and intensities of X-rays leaving the sample. SEM produces images of a sample by scanning the surface with a focused beam of electrons and gives information about the surface topography and composition of the sample. The diversity and ambiguity of green-synthesized materials necessitate the use of multiple techniques for valid characterizations. In our study, the synthesized catalytically active organic-inorganic composite gCuHCF was examined using FTIR, DLS, XRD and SEM (see Section 2.4).

#### 3.2.1. FTIR Characterization

The FTIR spectrum of the sample is presented in Figure S2. The FTIR spectrum was described in detail in our previous work [40]; it demonstrates the presence of the following groups: O-H, N-H, C≡N, C-H, C-O, C-N, Fe-C≡N and H_2_O-Cu-CN. Hydroxyl groups were identified by the bands at 3456 and 3050 cm^−1^, which are related to O-H stretching vibrations; and at 1398 cm^−1^, which corresponds to O-H bending [57]. Amine groups were determined by the bands at 3437 and 2994 cm^−1^ (primary amine stretching) and at 1638 cm^−1^ (assigned to N-H bending) [57]. The 2875 cm^−1^ band was attributed to C-H stretching; the 1476, 790 and 719 cm^−1^ bands corresponded to C-H bending [57]. The signals at 1122 and 1109 cm^−1^ can be explained by stretching vibrations of C-O and C-N groups, respectively [57]. The presence of C≡N groups was confirmed by the band at 2105 cm^−1^, reflecting stretching vibrations of this group [58]. The bands in the fingerprint region in the 509–667 cm^−1^ range can be related to Fe-CN linear bending, and the band at 468 cm^−1^ to Fe-C stretching [58]. The 2010 cm^−1^ band indicates the presence of a H_2_O-Cu-CN moiety [58]. The results of FTIR showed the presence of copper cyanoferrate particles enveloped by an organic layer with hydroxyl and amine groups, probably of protein origin.

#### 3.2.2. DLS Studies

The main results of the DLS measurements were described in detail in our previous work [40]. The DLS demonstrated heterogeneous mean hydrodynamic diameters of the particles in a tested gCuHCF. It is worth mentioning that very large differences in hydrodynamic diameters were found for various dilutions of the sample. In the most concentrated sample, only one size fraction was detected. There were probably larger agglomerates of particles in the concentrated suspensions that could not be measured by the designated instrument since the upper limit of measurement was 10,000 nm. After dilutions under gentle agitation, large aggregates disintegrated, and two fractions of particles were obtained.

In concentrated suspensions, the hydrodynamic diameter in the smaller particle fraction was 445 nm, whereas after dilution of the sample, two fractions were detected. The polydispersity index exceeded 10% for all dilutions. This result proved that the tested sample was not monodispersed. The zeta-potential was negative, estimated as −20.9 mV. This value characterizes the suspension state of gCuHCF as the threshold of delicate dispersion.

Particle concentration and mean size of the gCuHCF fraction, estimated by the particle counter, were 2.00 × 10^6^ mL^−1^ and 3.04 ± 1.98 μm, respectively.

#### 3.2.3. X-ray Diffraction (XRD) Analysis

The XRD pattern of the particles is shown in Figure S3. Diffraction peak positions and their relative intensities reflect the cubic crystalline structure of gCuHCF. Parameters of the crystal cell were calculated from the XRD pattern data (Table 2). The crystal cell belongs to a cubic type with the parameter a = 7.071 Å. Crystallite size was estimated as 156 ± 13 Å.

#### 3.2.4. SEM

**Figure 3:**
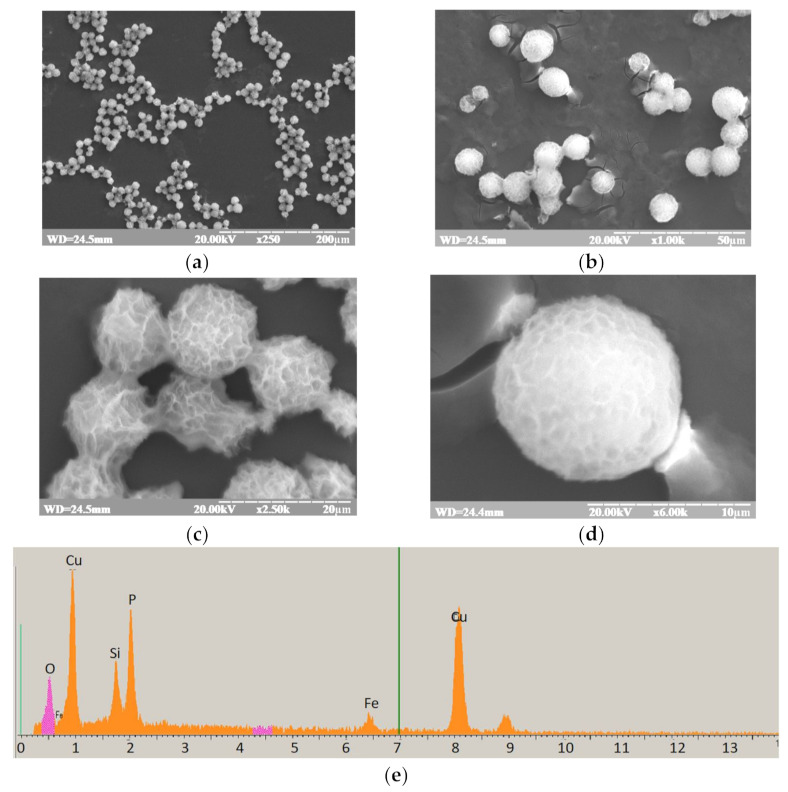
The results of gCuHCF study using SEM with RSM: (**a**–**d**)—SEM images at different magnifications; (**e**)—X-ray spectral characteristics.

gCuHCF was characterized by SEM coupled with X-ray microanalysis (SEM-XRM). It was found in our previous work [40] that SEM can supply information on the size, distribution, and shape of the tested sample. Figure 3a–d presents the overall morphology of the flower-like particles formed in the process. The XRM images of the gCuHCF film show the characteristic peaks for Cu and Fe (Figure 3e). According to the SEM results, the synthesized gCuHCFs are not nano-sized but rather microparticles.

Likewise, the different analytical approaches demonstrated that the synthesized gCuHCF is a suspension of micro-sized particles. These observations were confirmed by different methods: means of particle counting, dynamic light scattering, zeta-potential analysis, and SEM.

Based on the gCuHCF images presented in Figure 3, the studied catalytically active composite material may be described as “organic-inorganic micro/nanoflowers” (hNFs).

hNFs belong to a class of flower-like hybrid materials that self-assemble from metal ions and organic components, such as enzymes, DNA, and amino acids, into flower-like micro/nano superstructures [59,60]. hNFs are widely used for the development of stable, robust, reusable, efficient and cost-effective systems for the immobilization of biomolecules. Some hNFs were shown to exhibit an intrinsic PO-like activity [61,62]. Due to their remarkable performance—the simplicity of their synthesis; their high surface area; excellent thermal, storage, and pH stability; and catalytic activity—hNFs have various potential applications in bioremediation, bioassays, biomedicine, industrial biocatalysis and wastewater treatment [60]. Promising results were reported for hNFs in biosensing, including electrochemical biosensors, colorimetric biosensors and point-of-care diagnostic devices [60,61,62].

### 3.3. Application of the gCuHCF as a PO Mimetic in Amperometric (Bio)sensors

The applicability of the gCuHCF as a chemo-sensor for H_2_O_2_ detection was demonstrated in our previous work [40]. Quantitative analysis of a real sample of commercial disinfectant was carried out. The average H_2_O_2_ concentration determined by the gCuHCF-based chemo-sensor was shown to be well correlated with the manufacturer’s data, with an error of less than 10%.

#### 3.3.1. Properties of gCuHCF

**Figure 4:**
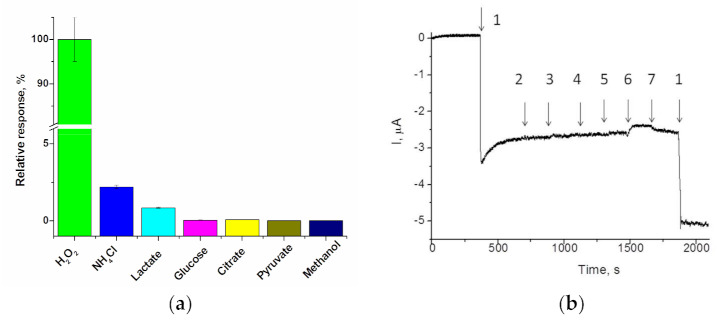
The selectivity tests for gCuHCF/GE: (**a**)—current responses in relative units (%), on the added analytes up to 2 mM concentration, as a ratio of the detected signals to the value of the highest current response; (**b**)—chronoamperograms as outputs on the added analytes (1–7) up to 0.5 mM concentration: (1)—H_2_O_2_, (2)—glucose, (3)—glycerol, (4)—methanol, (5)—sodium citrate, (6)—sodium lactate, (7)—ammonium chloride. Conditions: working potential −50 mV vs. Ag/AgCl (reference electrode), 50 mM NaOAc buffer, pH 4.5 at 23 °C.

Selectivity of the ABS towards the target analyte is of great importance, especially for the analysis of real samples. In this paper, to study the selectivity of the gCuHCF, a modified GE was tested for its ability to respond to a number of analytes: glucose, alcohols, organic acids, and ammonium ions, etc. The selectivity of the constructed chemo-sensor was estimated for the individual natural substrates (Figure 4a) as well as for their mixture with hydrogen peroxide (Figure 4b). The results presented in Figure 4b demonstrate that the presence of various compounds in the analyzed mixture does not interfere with H_2_O_2_ determination.

The amperometric analysis was performed using CV and chronoamperometry at different potentials (−50 and +150–200 mV) in different buffer solutions, with a pH from 4.0 to 8.0 (data not shown). It was demonstrated that neither methanol, glycerol, organic acids, nor glucose elicited any signals, while hydrogen peroxide (at −50 mV), ammonium ions and L-lactate (both at +200 mV) were found to elicit significant current responses on the gCuHCF/GE under the tested conditions. Current responses to L-lactate and ammonium under the potential −50 mV were insignificant (Figure 4).

Moreover, we demonstrated that in gCuHCF formation, Fc*b*_2_ was concentrated from the diluted solutions due to co-precipitation with the gCuHCF-based hNFs. When immobilized on a GE, the gCuHCF may become an ABS for L-lactate. CV analysis showed that the current output due to the L-lactate addition correlated with Fc*b*_2_ activity in the sensing layer (data not shown). Thus, the proposed method of hNF formation, using oxido-reductase in the presence of its substrate, may be a promising platform for the concentration and stabilization of any enzyme.

Additionally, using a laccase as a model oxidase, we demonstrated that the gCuHCF not only displayed enzymatic (PO) activity but also an electro-mediator ability (data not shown).

Preliminary experiments for the development of biosensors for primary alcohols and L-amino acids (based on alcohol oxidase and L-amino acid oxidase, respectively) were carried out (data not shown). The obtained results indicated that the gCuHCF and other gHCFs have a potential for use as PO-like composites for the construction of amperometric biosensors with any oxidase.

We conclude that the gCuHCF that was obtained with Fc*b*_2_ assistance, forming a flower-like micro-superstructure, is a prospective organic-inorganic composite material for biosensor construction. It is a stable, catalytically and electrochemically active carrier for enzyme concentration, immobilization and stabilization.

#### 3.3.2. Optimization of H_2_O_2_ Sensing

To improve the conditions for exploiting the biosensor, the optimal buffer, pH and working potential were estimated. For optimization of the chemo-sensor and further biosensor construction, the quantity of gCuHCF material on the surface of the GE, as well as the enzyme/gCuHCF ratio, were determined experimentally.

We analyzed the correlation of PO-mimetic activity with the effectiveness of H_2_O_2_ sensing, using the gCuHCF/GE under different conditions of pH and working potential.

The dependence of the chemo-sensor’s analytical characteristics on the quantities of gCuHCF placed on the GE surface was studied under the working potential −50 mV in 50 mM NaOAc, pH 4.5. The results are presented in Figure S4 and are summarized in Table 3. Based on the data, the optimal PO-like activity of the gCuHCF for achieving the highest sensitivity under the described conditions is 2–5 mU.

**Figure 5:**
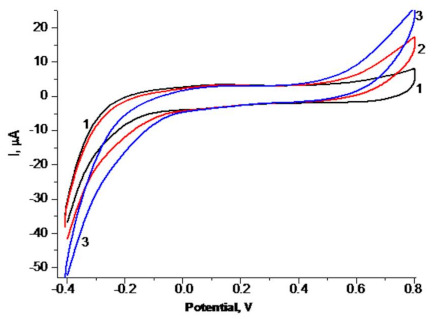
Cyclic voltammograms (CV) of the gCuHCF/GE. CV profiles (1–3) as outputs upon addition of H_2_O_2_ up to concentrations: (1)—0 mM (black); (2)—0.17 mM (red); (3)—0.5 mM (blue) mM. Conditions: scan rate 50 mV·s^−1^; Ag/AgCl (reference electrode) in 50 mM PB, pH 6.0. The sensing layer contains 0.35 mU of PO-like activity.

The optimal working potential for H_2_O_2_ sensing was determined using a CV study (Figure 5), followed by chronoamperometry experiments at pH 6.0 (data not shown). The decision to change the conditions of the experiments, and work under a pH range of 6–8, was necessitated by our plans to develop biosensors using different oxido-reductases. Many microbial enzymes have shown optimal activity near these pH values.

As seen in Figure 5, the optimal working potentials for H_2_O_2_ sensing under pH 6.0 were lower than −100 mV. To select the best conditions for achieving the highest gCuHCF/GE sensitivity, we determined its analytical parameters under different potentials, namely, −50 and −200 mV (Figure S5). According to the data, the chemo-sensor sensitivity under −200 mV was 2.7-fold higher than under −50 mV.

#### 3.3.3. Development of an Amperometric Biosensor for Glucose Determination

In our previous work [40], we reported on the construction of a mono-enzyme amperometric biosensor (ABS) for glucose, using gCuHCF as the PO mimetic and commercial glucose oxidase (GO). It is worth mentioning that the control gCuHCF/GE did not show any amperometric output in response to glucose. The sensitivity of the developed GO/gCuHCF/GE was rather low (76 A·M^−1^·m^−2^). In the current study, we set a goal to develop an improved GO/gCuHCF/GE with elevated/optimized analytical characteristics. We carried out the investigation of the gCuHCF as an artificial PO in more detail by studying the influence of various experimental stages on the effectiveness of H_2_O_2_ sensing; we describe these results in Section 3.3.2. The next task was the optimization of glucose biosensing.

**Figure 6:**
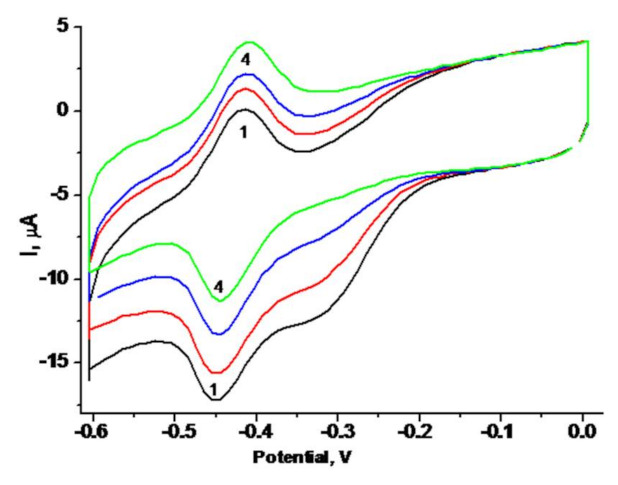
Cyclic voltammograms (CV) of the GO/gCuHCF/GE. CV profiles (1–4) as outputs upon addition of glucose up to concentrations: (1) 0, (2) 0.17, (3) 0.5, (4) 1.3 mM. Conditions: scan rate 50 mV·s^−1^; Ag/AgCl (reference electrode) in 50 mM PB, pH 6.5. The sensing layer of the biosensor contains 0.5 mU of PO-like gCuHCF and 40 mU of GO.

According to Figure 6, the optimal working potential for glucose sensing determined via CV measurement was −450 mV. However, to avoid a possible interference of various substances on the electrode response in the presence of oxygen at high voltage, we chose a lower working potential, namely, −250 mV. This requirement is relevant for the application of the biosensor for the analysis of real samples, e.g., food products.

For optimization of the biosensor composition, the enzyme/gCuHCF ratio on the GE surface was determined experimentally (data not shown). It was found that the optimal ratio, calculated from total activities (GO and PO-like gCuHCF), was 80. Activities of the GO and gCuHCF were estimated with o-dianisidine, as described in Section 2.2 and Section 2.5, respectively.

**Figure 7:**
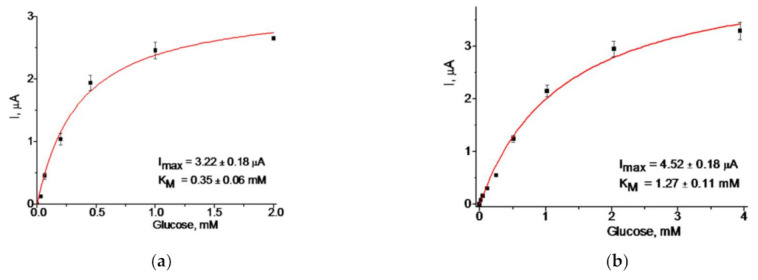

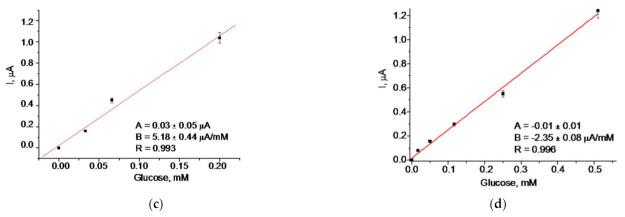
Characteristics of the GO/gCuHCF/GE under different working potentials: (**a**,**b**)—dependences of the current response on increasing concentrations for glucose determination; (**c**,**d**)—calibration graphs. Conditions: working potentials −250 (**a**,**c**) and −300 mV (**b**,**d**) vs. Ag/AgCl (reference electrode), 50 mM phosphate buffer, pH 6.0 at 23 °C. The GE contains 0.5 mU of PO-like activity and 40 mU GO.

Figure 7 demonstrates the best results obtained from the constructed GO-ABSs. To select the optimal working potential for GO-ABS exploitation, we estimated its analytical parameters under two potentials, at −250 and at −300 mV (Figure 7). Taking into account the parameters (b) from the linear regression graphs (Figure 7b,d) and the square of the electrode surface (7.3 mm^2^), we calculated the sensitivities of the GO-ABS to glucose. These and other analytical characteristics of the developed GO/gCuHCF/GEs are summarized in Table 4. According to Table 4, the sensitivity (A M^−1^m^−2^) at the potential −250 mV was 2.2-fold higher than at −300 mV, and 9.4-fold higher than at −50 mV. Thus, −250 mV was chosen as the optimal working potential for the exploitation of a GO/gCuHCF-based ABS.

Thus, we determined the optimal conditions for construction and exploitation of the most effective and highly sensitive GO-based ABS: the ratio of GO activity to PO-like activity of gCuHCF was shown to be 80 under conditions of −250 mV working potential, 50 mM phosphate buffer, and pH 6.0.

#### 3.3.4. Testing of GO/gCuHCF/GE Biosensor for Glucose Analysis in Juice Samples

In order to demonstrate the practical feasibility of the constructed ABS, the developed biosensor was used for glucose analysis in three fruit juice samples using the graphical method known as the standard addition test (SAT). Graphical SAT is a type of quantitative analysis often used in analytical chemistry when a standard is added directly to the aliquots of the analyzed sample. SAT is used in situations where sample components also may contribute to the analytical signal, which makes it impossible to use routine calibration methods. Estimation of glucose concentration in the initial sample was performed using the equation C = AN/B, where A and B are parameters of a linear regression and N is the dilution factor.

**Figure 8:**
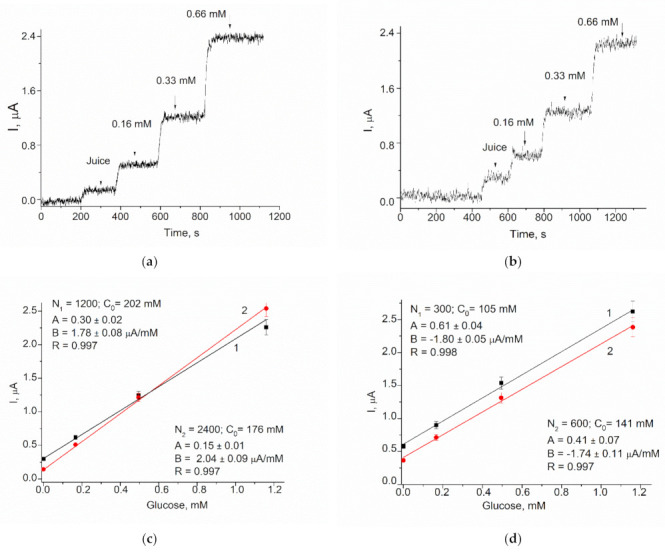
The example of glucose analysis using the biosensor in samples of juices: Multivitamin “Sadochok” (**a**–**c**), and apple–pear “Galicia” (**d**), in two dilutions; chronoamperograms (**a**,**b**), and corresponding linear graphs (**c**,**d**). Conditions: working potential −250 mV vs. Ag/AgCl (reference electrode), 50 mM phosphate buffer, pH 6.0 at 23 °C.

Figure 8 demonstrates in detail the algorithm of glucose estimation using two juices as the examples. The results of glucose determination in the juices sampled by the proposed biosensor and by a commercial enzymatic kit are presented in Table 5. The average glucose concentrations determined from the data in Figure 8 differ by less than 10% from the data obtained using the reference method (Table 5).

## 4. Conclusions

In the current research, we report the development of reagentless amperometric H_2_O_2_-sensitive sensors with artificial peroxidases (PO). As PO mimetics, “green” hexacyanoferrates (gHCFs) of transition and noble metals were used, which were synthesized via the oxidoreductase Fc*b*_2_. The gCuHCF was identified as the most effective PO mimetic and was characterized in detail concerning its structural, catalytic and electrochemical properties.

SEM analysis demonstrated that the gCuHCF formed a flower-like micro/nano superstructure. Thus, it may be used not only as a H_2_O_2_-sensitive platform for the development of oxidase-based biosensors but also as a carrier for enzyme concentration, immobilization and stabilization.

An amperometric glucose-oxidase-based biosensor with gCuHCF as the PO mimetic was developed. It exhibited high sensitivity (710 A M^−1^m^−2^), a broad linear range and good selectivity. The practical feasibility of the constructed biosensor was demonstrated on samples of fruit juices.

The obtained results indicated that the gCuHCF and other gHCFs may have a potential for use as PO-like composites for the construction of amperometric biosensors with any oxidase.

## Figures and Tables

**Table 1:** Comparative analytical characteristics of HCFs as artificial peroxidases on graphite electrodes.

Sensitive Film	*K_M_^app^*, mM	*I_max_*, µA	Linear Range, Up to, mM	Sensitivity, A M^−1^m^−2^
gCuHCF	31.0 ± 4.4	138.0 ± 8.5	0.8	1620
gPB	8.0 ± 1.1	27.8 ± 1.0	0.4	1090
gPdHCF	33.1 ± 3.9	62.4 ± 3.0	0.8	697
gCeHCF	3.5 ± 0.4	27.3 ± 0.8	3.2	560
PO	4.9 ± 1.1	5.0 ± 0.2	0.4	352
gYHCF	10.1 ± 0.9	21.6 ± 1.1	3.1	214
gCoHCF	9.3 ± 0.9	17.2 ± 1.0	0.8	159
chCuHCF	20.0 ± 3.5	6.5 ± 0.4	0.8	110
gMnHCF	92.3 ± 15.2	21.1 ± 2.1	0.8	98
gZnHCF	25.5 ± 2.2	4.0 ± 0.2	6.5	22
gNdHCF	21.3 ± 1.7	3.1 ± 0.1	6.5	16
gCdHCF	40.0 ± 5.4	2.6 ± 0.2	1.5	15

**Table 2:** Crystal cell parameters of gCuHCF.

Characteristics	Data
Crystal System	Cubic
Space group	Fm-3m (225)
Parameter of cell	a = b = c = 7.071 Å V = 250.00 Å^3^
Crystal	Centrosymmetric
Pearson Symbol	cF 60.02
ANX	AB2C6X6
Molecular Weight	226.08 g/mol
Structural Density	2.25 g/cm^3^

**Table 3:** Effect of gCuHCF PO-mimetic activity on the analytical characteristics of the modified GEs at pH 4.5.

Number	gCuHCF	Placed on GE	Sensitivity, A M^−1^m^−2^	*I_max_*, µA	*K_M_^app^*, mM
	Volume, µL	Activity, mU			
1	0.5	1	261	59.0 ± 3.6	33.3 ± 4.5
2	1	2	1065	162.3 ± 20.7	54.8 ± 13.4
3	2.5	5	747	114.6± 12.7	22.4 ± 5.17
4	5	10	139	66.6 ± 13.9	22.4 ± 9.69

**Table 4:** Analytical characteristics of the developed GO/gCuHCF/GEs.

Number	Composition of Sensing Film		Voltage, mV	Sensitivity, A M^−1^m^−2^	*I_max_*_,_ μA	Linear Range, Up to μM	*K_M_^app^*, mM
	GO, mU	PO Mimic, mU					
1	300	20	−50	76	1.15	3000	1.8
2	40	0.5	−250	710	3.22	200	0.35
3	40	0.5	−300	322	4.52	500	1.3

**Table 5:** Results of glucose estimation in the samples of fruit juices.

Juice	Glucose, mM		
	Biosensor	Reference	Difference, %
Multi vitamin, “Sadochok”	189 ± 17	206 ± 15	8.6
Apple–pear, “Galicia”	123 ± 10	131 ± 12	6.3
Apple fresh	186 ± 16	202 ± 18	8.2

## Data Availability

Data is contained within the article: Gayda, G.; Demkiv, O.; Gurianov, Y.; Serkiz, R.; Gonchar, M.; Nisnevitch, M. 2020. “Green” nanozymes: synthesis, characterization and application in amperometric (bio)sensors. Proceedings 60(1), 58; doi.org/10.3390/IECB2020-07072 and Supplementary Material section.

## Supplementary Materials

The following are available online at https://www.mdpi.com/article/10.3390/bios11060193/s1, Figure S1. Amperometric characteristics of the several modified electrodes: chronoamperograms (left), dependence of the current response on increasing concentrations of H_2_O_2_ (middle), and calibration graphs (right); H_2_O_2_-sensing films are the following: gPdHCF (a); gCeHCF (b); gYHCF (c); gCoHCF (d); gMnHCF (e); gZnHCF (f); gNdHCF (g); gCdHCF (h) and chCuHCF (i). Conditions: working potential −50 mV vs. Ag/AgCl (reference electrode), 50 mM NaOAc buffer, pH 4.5 at 23 °C. Figure S2. FTIR spectrum of the gCuHCF. Figure S3. DLS plots of particle hydrodynamic diameter of the sample at various concentrations: the green line represents a 1.3 × 10^8^ mL^−1^, the yellow line 6.6 × 10^7^ mL^−1^, and the red line 3.3 × 10^7^ mL^−1^ particle concentration. Figure S4. X-ray diffraction analysis of the gCuHCF’s synthesized particles. Figure S5. Effect of the PO-mimetic activity on the efficiency of H_2_O_2_ sensing: current response to increasing concentrations of H_2_O_2_ (a–e); and calibration graphs (f) for the GEs modified with different quantities of gCuHCF: (a) 1 mU, (b) 2 mU, (c) 5 mU, (d) 10 mU, (e,f); combined graph lines (1–4) correspond to graphs (a–d), respectively. Conditions: working potential −50 mV, Ag/AgCl (reference electrode) in 50 mM NaOAc, pH 4.5. Figure S6. Effect of PO-mimetic activity and working potential on analytical characteristics of the gCuHCF/GE: current responses to increasing concentrations of H_2_O_2_ (a,c); and the corresponding calibration graphs (b,d) for the GE modified with different quantities of gCuHCF: (1)—0.07 mU, (2)—0.15 mU, (3)—0.40 mU. Conditions: working potential −50 mV (a,b) and −200 mV (c,d), Ag/AgCl (reference electrode) in 50 mM phosphate buffer, pH 6.0.

## Abbreviations

ABTS	2,2′-azinobis (3-ethylbenzothiazoline-6-sulfonate) diammonium salt
chHCF	Chemically synthesized HCF of a transition metal
CV	Cyclic voltammetry
DLS	Dynamic light scattering
Fc*b*_2_	Flavocytochrome *b*_2_
FTIR	Fourier transform infrared spectroscopy
gHCF	Green-synthesized hexacyanoferrate of a transitional or noble metal
gHCF/GE	Green-synthesized hexacyanoferrate immobilized on GE
gNPs	Green-synthesized NPs
GE	Graphite electrode
GO	Glucose oxidase
gPBA	Green synthesized Prussian blue analog
HCF	Hexacyanoferrate of a transitional or noble metal
hNFs	Organic-inorganic hybrid nanoflowers
*I_max_*	Maximal current response on tested analyte at substrate saturation
*K_M_^app^*	Apparent Michaelis–Menten constant
NaOAc	Sodium acetate buffer
NZ	Nanozyme
NP	Nanoparticle
PAAG	Polyacrylamide gel
PB	Prussian blue
PBA	PB analog
PO	Peroxidase
SAT	Standard addition test
SEM-XRM	Scanning electron microscopy coupled with X-ray microanalysis
XRD	X-ray diffraction analysis
