# Supplementary material for: “Green” Prussian Blue Analogues as Peroxidase Mimetics for Amperometric Sensing and Biosensing
Source: Biosensors (Basel). 2021 Jun 10;11(6):193. doi: 10.3390/bios11060193 (PMC8229941; doi:10.3390/bios11060193)
Supplement: Supplementary file 1 [file biosensors-11-00193-s001.zip › biosensors-1224179-supplementary.pdf]

Supporting information

"Green" Prussian Blue Analogues as Peroxidase Mimetics for Amperometric Sensing and Biosensing

Galina Z. Gayda [1,*], Olha M. Demkiv [1,2], Yanna Gurianov [3], Roman Ya. Serkiz [1], Halyna M. Klepach [4], Mykhailo V. Gonchar [1,4] and Marina Nisnevitch [3,*]

[1] Institute of Cell Biology, National Academy of Sciences of Ukraine, 79005 Lviv, Ukraine; galina.gayda@nas.gov.ua (G.G.); demkivo@nas.gov.ua (O.D); rserkiz@gmail.com (R.S.); gonchar@cellbiol.ua (M.G.)
[2] Faculty of Veterinary Hygiene, Ecology and Law, Stepan Gzhytskyi National University of Veterinary Medicine and Biotechnologies, 79000 Lviv, Ukraine
[3] Department of Chemical Engineering, Ariel University, Kyriat-ha-Mada, Ariel 4070000, Israel; yannag@ariel.ac.il (Y.G.); marinan@ariel.ac.il (M.N.)
[4] Department of Biology and Chemistry, Drohobych Ivan Franko State Pedagogical University, 82100 Drohobych, Ukraine (H.K.)
* Correspondence: galina.gayda@gmail.com; galina.gayda@nas.gov.ua; Tel.: +380-226-2144 (G.G.) and marinan@ariel.ac.il; Tel.: +972-3914-3042 (M.N.)

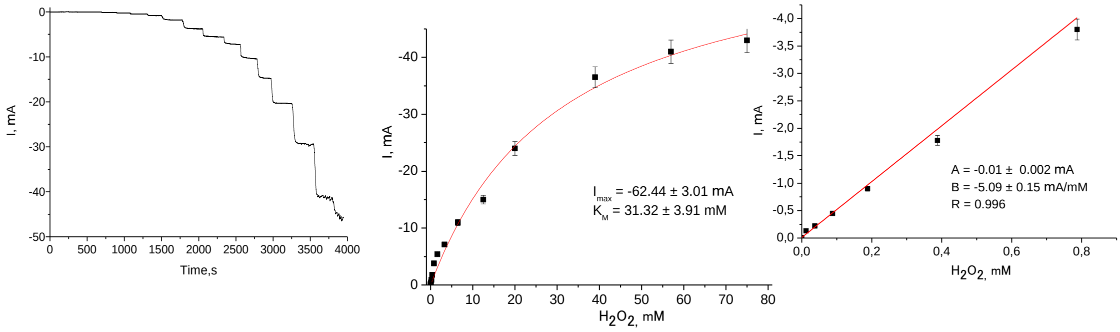
I, mA
Time,s
I, mA
H2O2, mM
Imax = -62.44 ± 3.01 mA
KM = 31.32 ± 3.91 mM
I, mA
H2O2, mM
A = -0.01 ± 0.002 mA
B = -5.09 ± 0.15 mA/mM
R = 0.996

(a)

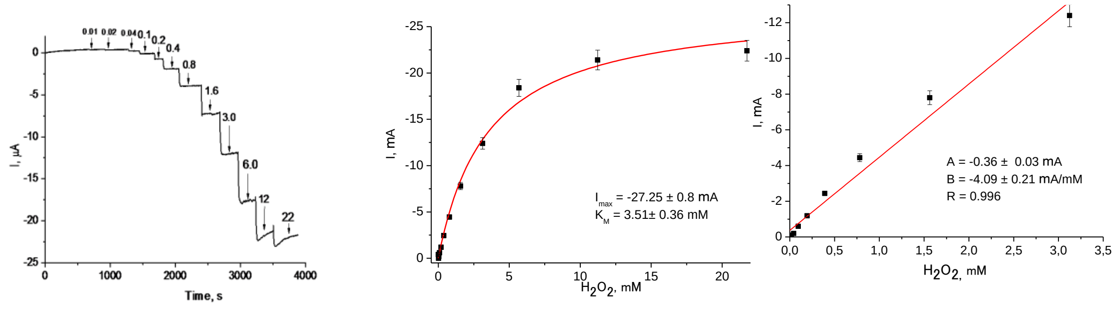
0.01 0.02 0.04 0.1 0.2 0.4 0.8 1.6 3.0 6.0 12 22
I, µA
Time, s
I, mA
H2O2, mM
Imax = -27.25 ± 0.8 mA
KM = 3.51± 0.36 mM
I, mA
H2O2, mM
A = -0.36 ± 0.03 mA
B = -4.09 ± 0.21 mA/mM
R = 0.996

(b)

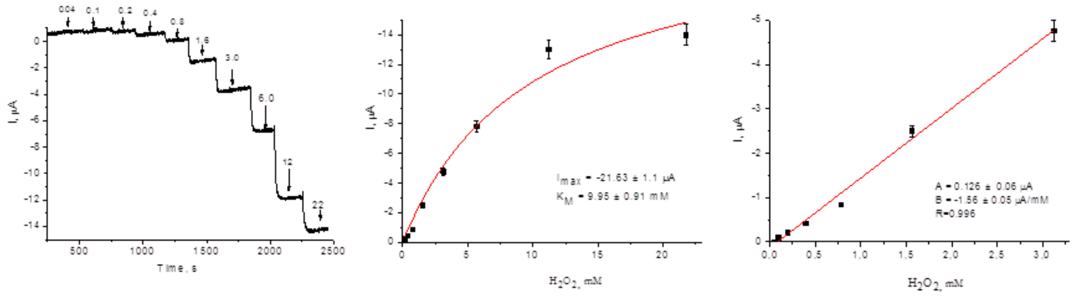
0.04 0.1 0.2 0.4 0.8 1.6 3.0 6.0 12 22
I, µA
Time, s
I, µA
H2O2, mM
Imax = -21.63 ± 1.1 µA
KM = 9.95 ± 0.91 mM
I, µA
H2O2, mM
A = 0.126 ± 0.06 µA
B = -1.56 ± 0.05 µA/mM
R=0.996

(c)

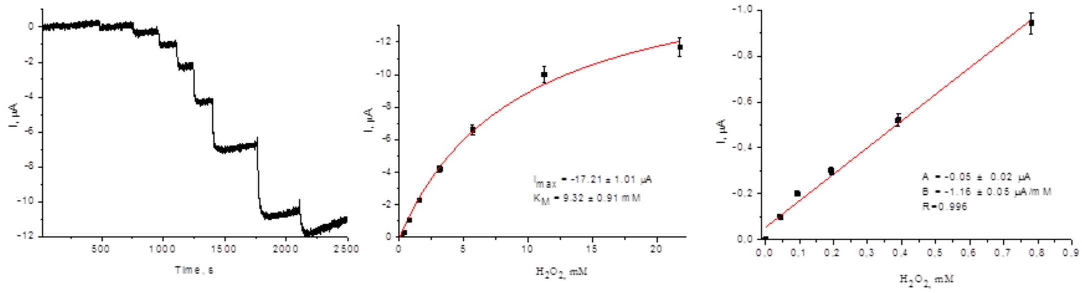
I, µA
Time, s
I, µA
H2O2, mM
Imax = -17.21 ± 1.01 µA
KM = 9.32 ± 0.91 mM
I, µA
H2O2, mM
A = -0.05 ± 0.02 µA
B = -1.16 ± 0.05 µA/mM
R=0.996

(d)

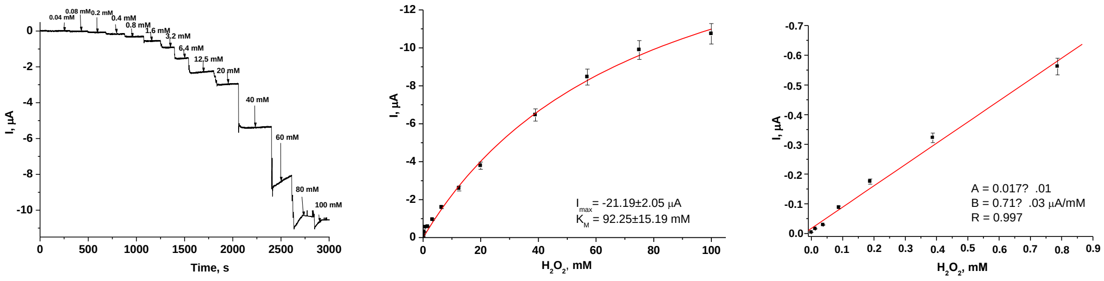
0.04 mM 0.08 mM 0.2 mM 0.4 mM 0.8 mM 1,6 mM 3,2 mM 6,4 mM 12,5 mM 20 mM 40 mM 60 mM 80 mM 100 mM
I, µA
Time, s
I, µA
H2O2, mM
Imax = -21.19±2.05 µA
KM = 92.25±15.19 mM
I, µA
H2O2, mM
A = 0.017? .01
B = 0.71? .03 µA/mM
R = 0.997

(e)

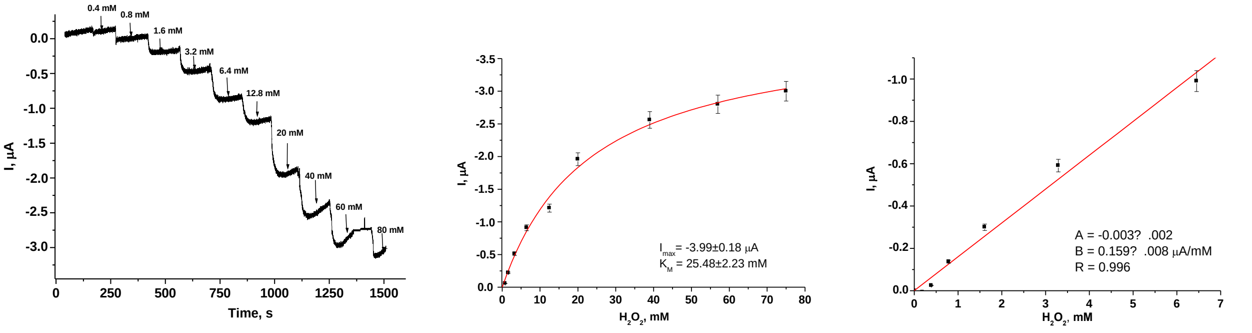

(f)

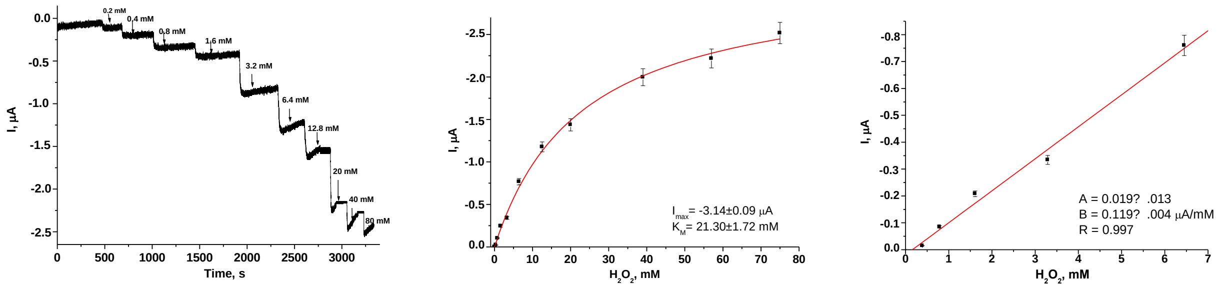

(g)

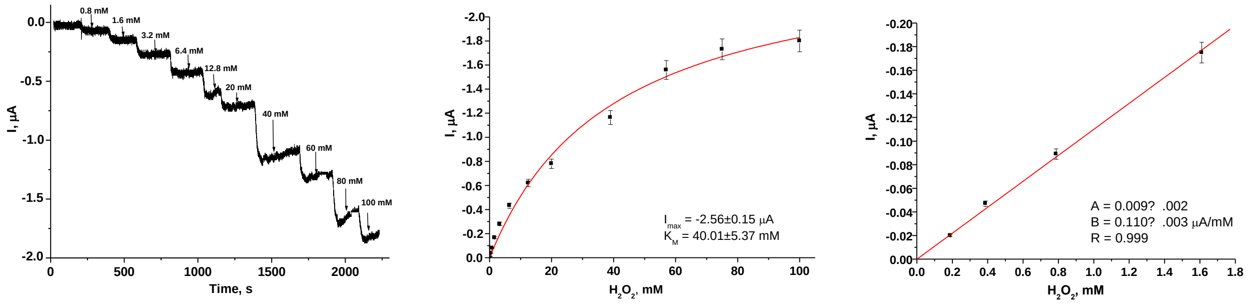

(h)

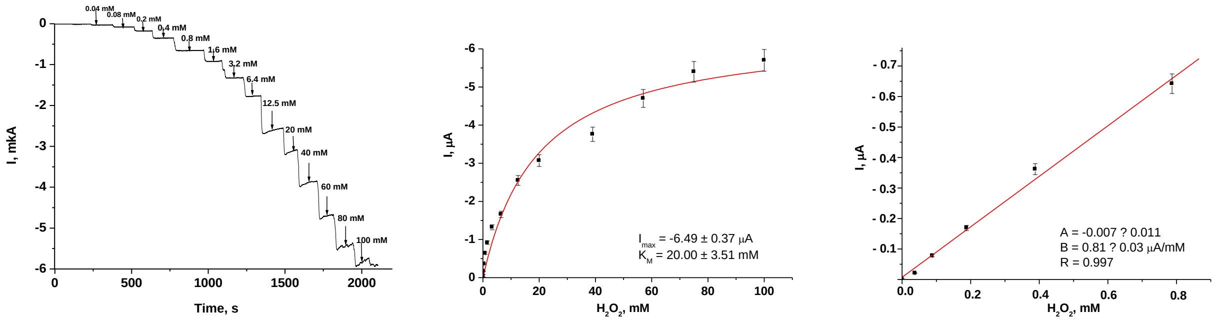

(i)

Figure S1. Amperometric characteristics of the several modified electrodes: chronoamperograms (left), dependence of the current response on increasing concentrations of H_2O_2 (middle) and calibration graphs (right). H_2O_2-sensing films are the following :gPdHCF (**a**); gCeHCF (**b**); gYHCF (**c**); gCoHCF (**d**); gMnHCF (**e**); gZnHCF (**f**); gNdHCF (**g**); gCdHCF (**h**) and chCuHCF (**i**). Conditions: working potential -50 mV vs. Ag/AgCl (reference electrode), 50 mM NaOAc buffer, pH 4.5 at 23°C.

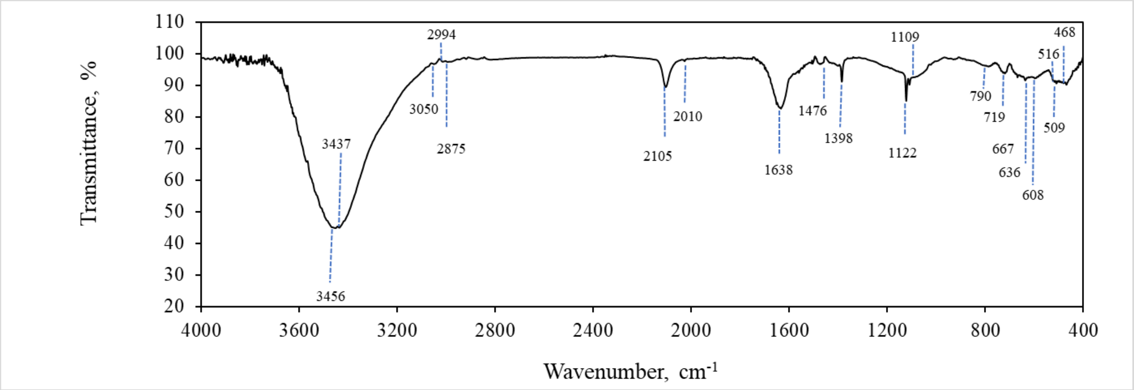

Figure S2. FTIR spectrum of the gCuHCF.

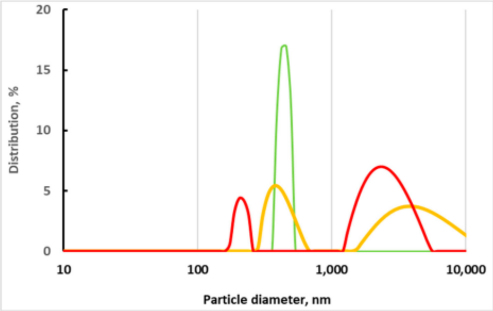

Figure S3. DLS plots of particle hydrodynamic diameter of the sample at various concentrations: the green line represents a 1.3×10^8 mL^{-1}, the yellow line 6.6×10^7 mL^{-1}, and the red line 3.3×10^7 mL^{-1} particle concentration.

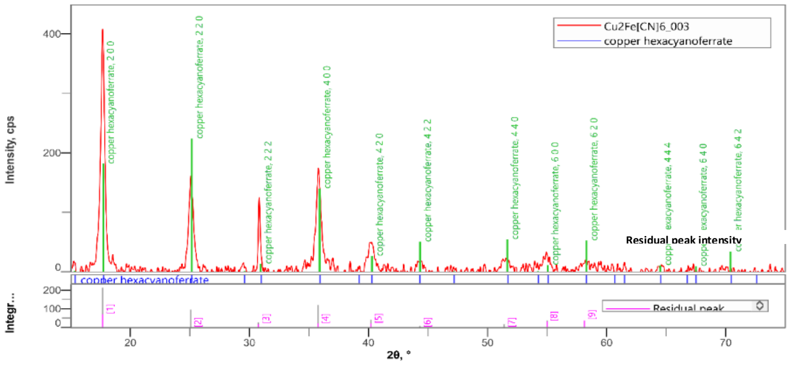

Figure S4. X-ray diffraction analysis of the gCuHCF synthesized particles.

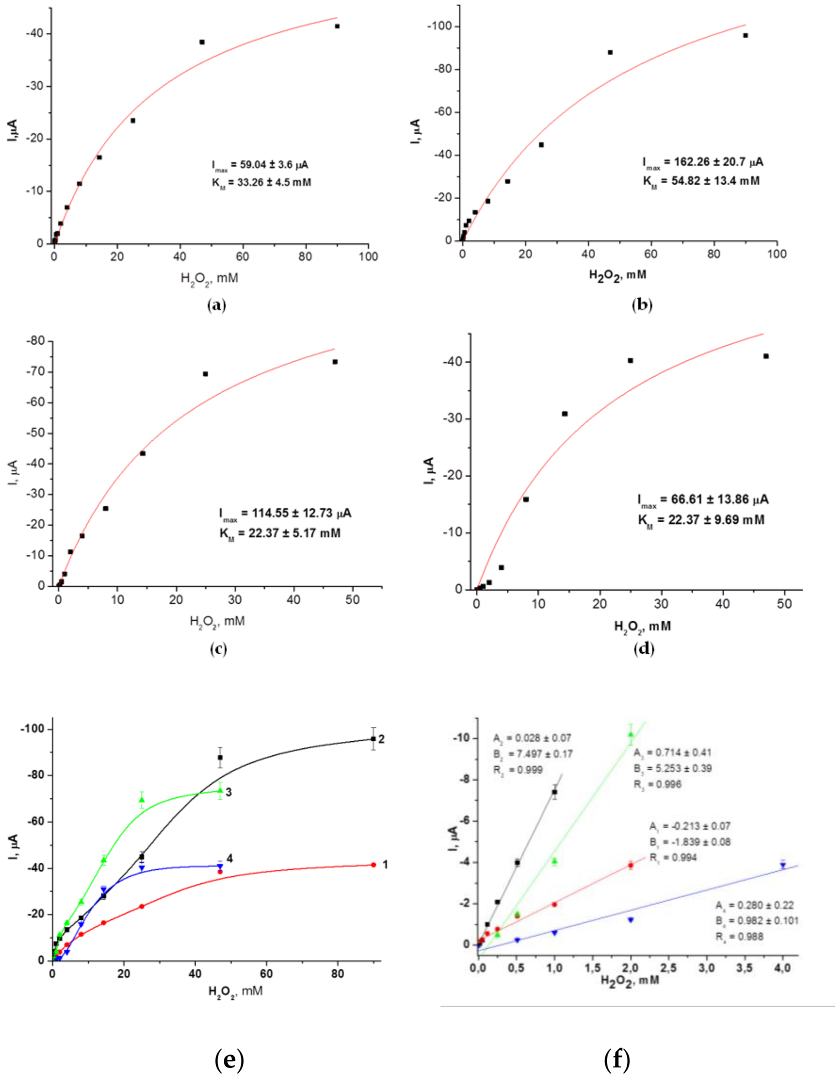

Figure S5. Effect of the PO mimetic' activity on the efficiency of H_2O_2 sensing. Current response on increasing concentrations of H_2O_2 (**a**-**e**) and calibration graphs (**f**) for the GEs modified with different quantities of gCuHCF: (**a**) 1 mU, (**b**) 2 mU, (**c**) 5 mU, (**d**) 10 mU, (**e**, **f**) – combined graphs, lines (1-4) correspond to graphs (**a**–**d**), respectively. Conditions: working potential –50 mV, Ag/AgCl (reference electrode) in 50 mM NaOAc, pH 4.5.

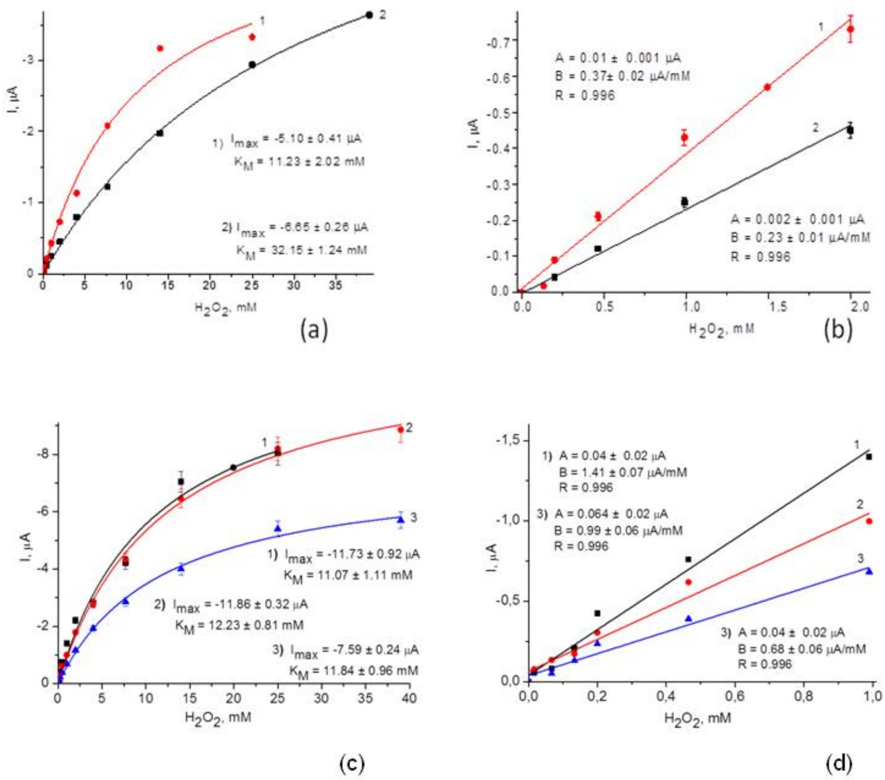

Figure S6. Effect of PO mimetic activity and working potential on analytical characteristics of the gCuHCF/GE. Current responses on increasing concentrations of H_2O_2 (**a**, **c**) and the correspondent calibration graphs (**b**, **d**) for GE, modified with different quantities of gCuHCF: (1) 0.07 mU, (2) 0.15 mU, (3) 0.40 mU. Conditions: working potential –50 mV (**a**, **b**) and –200 mV (**c**, **d**), Ag/AgCl (reference electrode) in 50 mM phosphate buffer, pH 6.0.
